# Periodontal and Caries Status in Relation to Oral Health Knowledge, Attitudes, and Behavior of Medical and Dental Students in Taxila, Pakistan

**DOI:** 10.7759/cureus.72718

**Published:** 2024-10-30

**Authors:** Maria Rabbani, Kinza Manzoor, Amna Faisal, Wajeeha Jabeen, Sarah Rabbani, Azka Haroon, Muhammad Kashif

**Affiliations:** 1 Community and Preventive Dentistry, Dental College, HITEC – Institute of Medical Sciences, Taxila, PAK; 2 Dental Biomaterials, Islamic International Dental College, Islamabad, PAK; 3 Community Dentistry, Dental College, HITEC – Institute of Medical Sciences, Taxila, PAK; 4 Oral Pathology, Bakhtawar Amin Medical and Dental College, Multan, PAK; 5 Oral Pathology, Dental College, HITEC – Institute of Medical Sciences, Taxila, PAK

**Keywords:** decayed missing and filled teeth (dmft) index, dental care, dental caries, oral health, periodontal status

## Abstract

Background

The importance of oral health in general well-being is widely acknowledged. It is expected that students belonging to dental and medical fields maintain a strong awareness of oral health and participate in public oral health awareness and initiatives, particularly in low-income nations such as Pakistan.

Objectives

The objective of the study was to assess the relation of periodontal status and dental caries status of medical and dental students in Taxila, Pakistan, with their oral health knowledge, attitude, and behavior.

Methodology

A cross-sectional study was conducted in Taxila targeting a total of 350 medical and dental students. Convenient sampling was used for the study. Data collection involved a structured questionnaire for oral health knowledge, attitudes, and behavior. The questionnaire was pilot tested, and Cronbach alpha was 0.70. Periodontal and dental caries was assessed using the Community Periodontal Index (CPI) and the Decayed, Missing, and Filled Teeth (DMFT) Index, respectively. Ethical approval and informed consent were obtained, maintaining confidentiality. Descriptive statistics summarized demographic and oral health data, whereas statistical tests explored associations of periodontal and dental caries status.

Results

Overall, the students’ mean percentage scores were 7.14 ± 1.5 on knowledge, 24.4 ± 3.5 on attitudes, and 7.53 ± 1.4 on behavior. The mean CPI score was 1.17, and the DMFT score was 1.64 ± 1.66. Notably, dental students were well aware of their oral health compared to medical students in all aspects. Analysis revealed that oral health knowledge and behavior were correspondent with good dental health, with significant results.

Conclusion

The medical and dental students of Taxila showed statistically significant oral health knowledge, positive attitudes, and behavior. The attitudes although alone did not significantly affect oral health status. However, enhancing oral health awareness among students requires collective efforts from health professionals.

## Introduction

The evolution of the concept of health, spurred by the World Health Organization's inclusion of social well-being, has led to a broader understanding of oral health. No longer limited to the absence of disease, oral health now encompasses factors contributing to overall well-being [1]. Daily activities such as eating, speaking, smiling, and societal contributions are recognized as determinants of an individual's well-being, underscoring the integral relationship between oral health and general well-being [1,2].

In both medicine and dentistry, there has been a paradigm shift from the traditional medical model to the socio-environmental model of health. This new perspective views health as the capability for optimal functioning, social, and psychological well-being. Within this framework, oral well-being is defined not only by the absenteeism of illness but also by the ability to maintain optimal oral function and aesthetics, thereby enabling individuals to fulfill their social roles [3,4].

The oral health concerns of individuals are intricately tied to their attitudes, which are shaped by personal experiences, societal perspectives, family traditions, and unique life events. These attitudes significantly influence oral health behaviors, with factors such as knowledge, beliefs, values, and skills playing pivotal roles. People who are informed and feel empowered about their oral health are more likely to practice good self-care habits [5,6].

Oral health promotion aims to disseminate knowledge beyond the dental profession, recognizing that empowering individuals with information is crucial for improving oral health outcomes. Dental caries and periodontal diseases stand as prevalent oral pathologies impacting populations across all age groups. In Pakistan, the prevalence of periodontitis is high, estimating at 37% in Punjab, 40% in Sindh, 20% in Khyber Pakhtunkhwa, and 3% in Baluchistan [7]. Risk behaviors such as frequent consumption of sugary foods and drinks, inadequate tooth brushing, smoking, alcohol use, and not visiting a dentist contribute to the development of these diseases. Such behaviors may begin in childhood or adolescence, as individuals gain autonomy and assume responsibility for their oral health [8,9].

Recent studies have shed light on the attitudes and behaviors of young adults concerning oral well-being, highlighting the link between knowledge, attitudes, behaviors, and overall oral health. For instance, research has shown that children with inadequate oral health knowledge are more susceptible to dental caries. However, there remains a gap in understanding the broader public's knowledge and attitudes toward oral diseases and prevention strategies [10-12].

Given that individuals with higher education backgrounds may grasp preventive concepts more readily, regardless of their field of study, professional college students present an ideal cohort for such investigations. Thus, the present study aims to assess the oral health knowledge, attitudes, behaviors, periodontal status, and dental caries status among medical and dental college students, contributing to deeper insights into oral well-being dynamics in this demographic.

## Materials and methods

Subject recruitment

The present study utilized a cross-sectional research design. The study was conducted in HITEC Institute of Medical Sciences Taxila affiliated with the National University of Medical Sciences Pakistan. The target population encompassed students from medical and dental fields enrolled in the institute from years 1 to 5. To ensure comprehensive representation across the years, a convenient sampling technique was employed. Using the Raosoft sample size calculator, the margin of error was set to 5% with 95% confidence level. The sample size calculated was 218 for medical students and 132 for dental students from the total population of 500 and 200, respectively. The collective sample size attributed to 350 students.

Data collection

The participants were asked to provide demographic information such as their specialty, grade, gender, and age. The questionnaire comprised 30 other questions aimed at assessing the oral health behavior, knowledge, and status of both dental and medical students (Appendix). The first section addressed oral health knowledge through 10 questions, exploring topics such as the signs of gum disease, tooth brushing, amount of toothpaste, prevention of tooth decay, and dental visits. The second and third sections focused on oral health attitudes and behavior and included 10 questions each, covering topics such as frequency and duration of tooth brushing, frequency of toothbrush replacement, dental visitation habits, methods of tooth brushing, and additional oral hygiene practices.

The questionnaire was reviewed by a panel of experts in dentistry and public health to ensure content validity. These experts assessed the relevance, clarity, and comprehensiveness of each question related to oral health knowledge, attitudes, and behaviors. Additionally, a pilot study was conducted with a sample of 30 students to assess the reliability of the questionnaire. Cronbach’s alpha was calculated to measure internal consistency, with a value of 0.7 indicating good reliability.

The survey was conducted using a paper-based format administered during in-person sessions at the institute. Students completed the questionnaire before undergoing clinical examination by trained dental professionals. The use of an offline, paper-based survey ensured that all students could participate, regardless of internet access.

After the questions, trained dental professionals, specialized in restorative dentistry and oral medicine, conducted examination using the Community Periodontal Index (CPI) [13] for periodontal status and the Decayed, Missing, and Filled Teeth (DMFT) index for dental caries status [14]. All examiners were dental professionals who underwent a calibration process prior to data collection. Calibration sessions were conducted to ensure consistency in clinical measurements using the CPI index and the DMFT index.

Ethical consideration

Before collecting data, ethical approval was secured from the Institutional Review Board (IRB) (Ref. No. Dental/HITEC/IRB/71; Dated: 01-04-2024), and all participants provided informed consent. Confidentiality and anonymity were rigorously maintained throughout the study process to uphold participants' privacy rights. Students were informed of the study’s objectives, their voluntary participation, and the right to withdraw at any time without any academic consequences. Anonymity was ensured by assigning each participant a unique code number, and personal data were kept confidential in compliance with ethical standards.

Data analysis

The data collected were examined using SPSS Version 29 (IBM Corp., Armonk, NY). Descriptive statistics were employed to summarize demographic characteristics, oral health knowledge, attitudes, and behaviors among the participants. Additionally, the chi-square test and t-test were applied to explore associations of periodontal status and dental caries status with oral health knowledge, attitudes, and behaviors. A p-value of less than 0.05 was considered statistically significant.

## Results

Student characteristics

A total of 350 students took part in the study, out of which 201 (57.4%) were males and 149 (42.6%) were females. The mean age of students was 20.89 ± 2.1 years, with a median age of 21 years. The students were separated into two categories in the light of their study field: 218 (62.3%) medical students and 132 (37.7%) dental students.

Knowledge, attitudes, and behavior of oral health

The mean score on knowledge of oral health was 7.14 ± 1.5 out of total score of 10. In terms of type of study, the mean score was 6.78 ± 1.6 in medical students and 7.73 ± 1.3 in dental students. The results showed statistical significance between the two variables, with a p-value less than 0.05. The thorough results of knowledge variables are demonstrated in Table 1.

**Table 1:** Knowledge of medical and dental students regarding oral health (n=350)

Questions		Correct		Wrong	
		n	%	n	%
Q1	General body well-being has relationship to oral well-being	300	85.7	50	14.3
Q2	Recommended brushing time	236	67.4	114	32.6
Q3	Regular brushing can protect from gum bleeding	269	76.9	81	23.1
Q4	Signs of gum disease	226	64.6	124	35.4
Q5	Purpose of fluoride in oral health	252	72.0	98	28.0
Q6	Recommended age for the first dental visit	232	66.3	118	33.7
Q7	Causes of tooth decay	260	74.3	90	25.7
Q8	Amount of toothpaste to use	235	67.1	115	32.9
Q9	Prevention of tooth decay	241	68.9	109	31.1
Q10	Prevent bleeding gum problems	249	71.1	101	28.9

The attitudes of students were assessed on a scale of 1 to 5, with 1 signifying solid understanding and 5 indicating solid conflict. The outcomes for all students are demonstrated in Table 2. In terms of overall attitude score, 297 (84.9%) showed a positive attitude, 31 (8.9%) showed a neutral attitude, and 22 (6.3%) showed a negative attitude towards oral health. The mean score was 24.4 ± 3.5.

**Table 2:** Attitudes of medical and dental students regarding oral health

Attitudes	1	2	3	4	5
I believe oral health is an important part of overall health	73 (20.9%)	144 (41.1%)	121 (34.6%)	12 (3.4%)	0 (0%)
Regular dental checkups are essential for maintaining good oral health	63 (18%)	140 (40%)	111 (31.7%)	33 (9.4%)	3 (0.9%)
Oral health problems can significantly impact a person's quality of life	63 (18%)	122 (34.9%)	101 (28.9%)	49 (14%)	15 (4.3%)
The general public's knowledge of oral health is very low	86 (24.6%)	144 (41.1%)	96 (27.4%)	21 (6%)	3 (0.9%)
I use floss daily	33 (9.4%)	81 (23.1%)	126 (36%)	100 (28.6%)	10 (2.9%)
Fluoride treatments are important in preventing cavities	76 (21.7%)	141 (40.3%)	95 (27.1%)	34 (9.7%)	4 (1.1%)
I am comfortable visiting the dentist for a routine checkup	44 (12.6%)	109 (31.1%)	105 (30%)	64 (18.3%)	28 (8%)
Cost is the biggest barrier to people seeking dental care	76 (21.7%)	147 (42%)	107 (30.6%)	18 (5.1%)	2 (0.6%)
There is a stigma associated with needing dental treatment	52 (14.9%)	111 (31.7%)	108 (30.9%)	63 (18%)	16 (4.6%)
My education has made me consider oral health to be important	66 (18.9%)	134 (38.3%)	114 (32.6%)	36 (10.3%)	0 (0%)

In terms of behavior of medical and dental students, the study found that a majority of users, comprising individuals 289 (82.5%), replaced their toothbrush within a three-month period. Additionally, in terms of the frequency of tooth brushing, 220 (62.8%) students stated that they brush their teeth twice daily. Among the participants, 276 (78.8%) individuals reported spending 1-2 minutes for brushing, which aligns with recommended guidelines.

Periodontal and dental caries status

**Figure 1:**
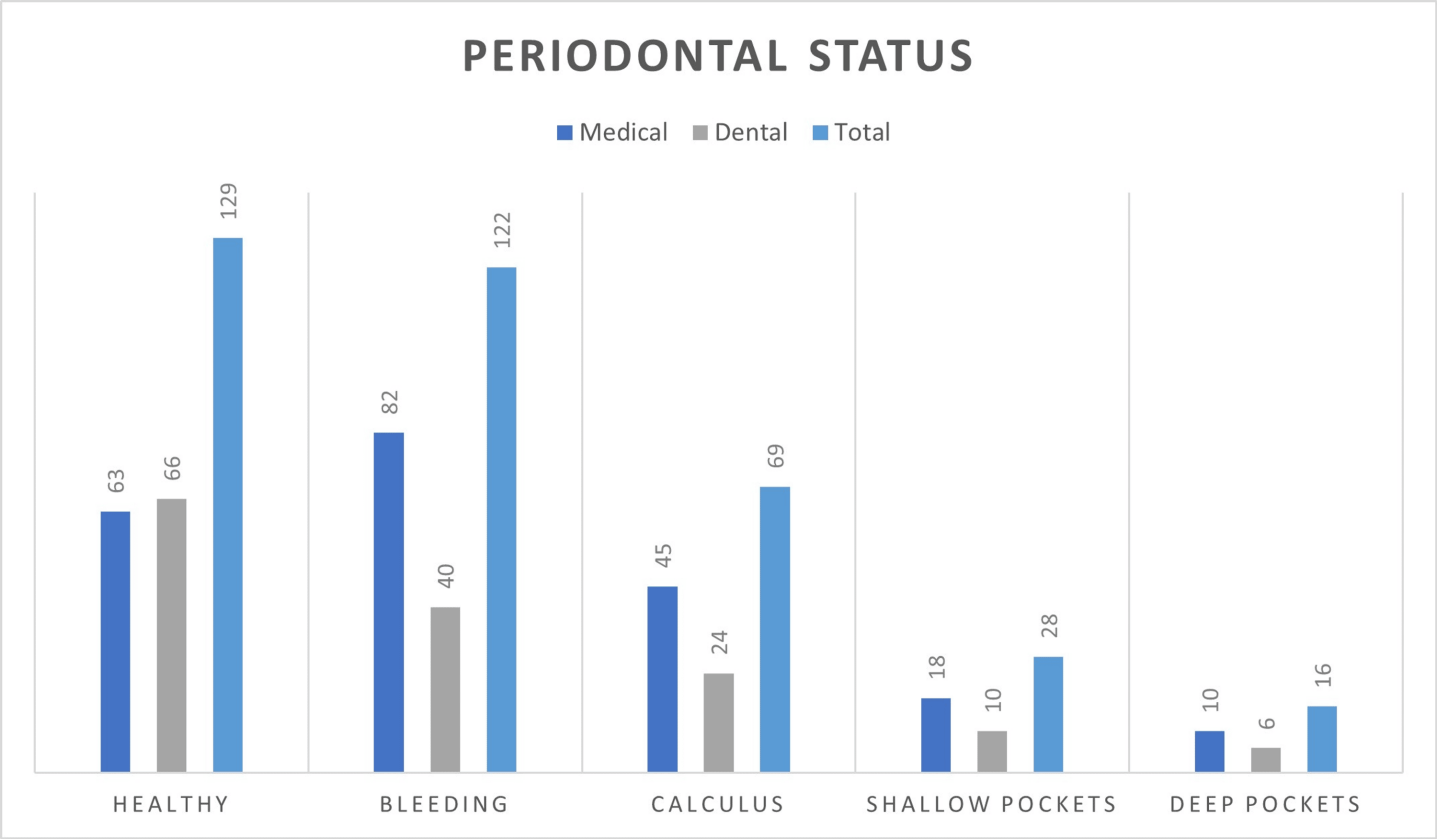
Periodontal status of study participants

The periodontal status of participants was assessed using the CPI. Each student’s highest score was used from their sextants. The index scores were categorized as follows: 0, healthy; 1, bleeding seen during testing; 2, calculus detected during probing, but no pocket greater than 3mm; 3, shallow pockets (4-5mm); 4, deep pockets (6mm or more). The results were statistically significant, with a p-value of less than 0.05 (Figure 1). The mean score was 1.22 ± 1.0 for medical students and 1.08 ± 1.1 for dental students.

**Figure 2:**
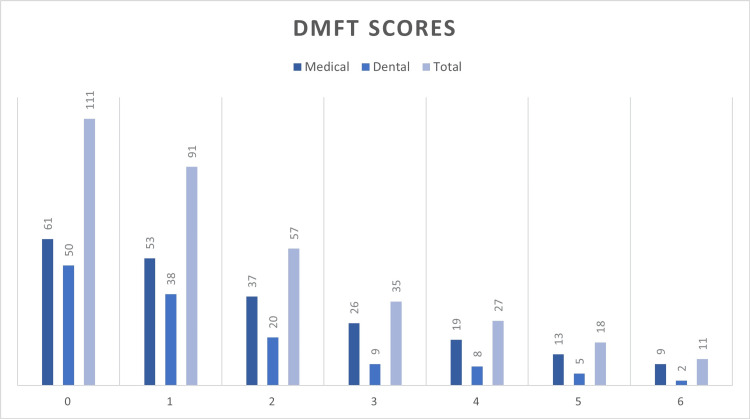
DMFT scores of study participants DMFT, Decayed, Missing, and Filled Teeth

For dental caries, the study participants were examined for DMFT score. The scores ranged from 0 to 6 (Figure 2), the results were statistically significant. The mean score of DMFT was 1.64 ± 1.66.

In terms of knowledge, attitudes, and behaviors with periodontal status, the study found that significant results in terms of all (Table 3).

**Table 3:** Relationship of oral health knowledge, attitudes, and behavior with periodontal and dental caries status *A p-value of less than 0.05 was considered significant (statistical test used: one-way ANOVA) CPI, Community Periodontal Index

Variables	CPI Scores	N	Mean	Standard Deviation	p-Value
Knowledge scores	Healthy	118	7.19	1.485	0.000*
	Bleeding	113	6.81	1.567	
	Calculus	76	7.41	1.618	
	shallow pockets	28	6.75	1.713	
	Deep pockets	15	8.67	0.617	
	Total	350	7.14	1.580	
attitudes scores	Healthy	118	24.71	3.972	0.041*
	Bleeding	113	24.58	3.414	
	Calculus	76	23.58	3.138	
	Shallow pockets	28	24.11	2.514	
	Deep pockets	15	26.33	3.638	
	Total	350	24.44	3.539	
Behavior scores	Healthy	118	7.43	1.304	0.00*
	Bleeding	113	7.05	1.375	
	Calculus	76	7.50	1.483	
	Shallow pockets	28	7.07	1.538	
	Deep pockets	15	8.67	0.617	
	Total	350	7.35	1.401	

The DMFT scores were divided into three categories: good, fair, and poor dental caries status. The results were compared with knowledge, attitudes, and behavior scores. The study found statistical significance between knowledge and behavior; however, the attitudes were not significant with DMFT (Table 4).

**Table 4:** Relationship between oral health knowledge, attitudes and behavior with DMFT score categories *A p-value of less than 0.05 was considered significant (statistical test used: one-way ANOVA) DMFT, Decayed, Missing, and Filled Teeth

Variables	DMFT Score Categories	N	Mean	Standard Deviation	p-Value
Knowledge scores	Good	202	7.00	1.569	0.007*
	Fair	57	7.74	1.357	
	Poor	91	7.08	1.662	
	Total	350	7.14	1.580	
Attitudes scores	Good	202	24.73	3.758	0.213
	Fair	57	24.07	3.380	
	Poor	91	24.04	3.080	
	Total	350	24.44	3.539	
Behavior scores	Good	202	7.27	1.360	0.044*
	Fair	57	7.77	1.337	
	Poor	91	7.25	1.495	
	Total	350	7.35	1.401	

## Discussion

This study provides an extensive assessment of oral well-being information, attitudes, behaviors, periodontal status, and dental caries status among medical and dental students in Taxila, Pakistan. The findings offer significant insights into the variances in oral well-being perspectives and conduct among these two groups and their implications on oral health outcomes.

The study found a notable variance in the oral health knowledge among medical and dental students, with dental students demonstrating significantly higher knowledge scores (mean score 7.73 ± 1.3) compared to their medical counterparts (mean score 6.78 ± 1.6). This is consistent with expectations given the specialized training dental students receive, which includes a more focused curriculum on oral well-being and cleanliness rehearses. The higher information scores among dental students likely contribute to better preventive behaviors and attitudes towards maintaining oral health. The overall high mean score of 7.14 ± 1.5 out of 10 across all students suggests a generally good level of oral health knowledge, but the disparity underscores the need for enhanced oral health education among medical students. Previous research supports this finding, indicating that dental students typically exhibit better oral health knowledge than their medical counterparts due to their specialized training. Given their role as healthcare professionals, their attitudes not only influence their own oral health behaviors but also possibly impact those of their patients and the community, and this finding correlates with similar studies [15,16]. In terms of gender, no significant differences were found between the variables. These discoveries really do contrast a few examinations that tracked down no distinctions in gender in oral well-being information and ways of behaving [17,18].

This positive outlook is encouraging as it suggests an elevated degree of mindfulness with respect to the significance of oral cleanliness. However, a small percentage (6.3%) exhibited negative attitudes, which could adversely affect their oral health practices and outcomes. These findings highlight the importance of fostering positive attitudes towards oral health, particularly in medical students, to ensure comprehensive healthcare practices. Previous studies have similarly noted that positive attitudes towards oral health are crucial for the adoption of good oral hygiene practices [19].

The periodontal status, assessed using the CPI, revealed that dental students had a slightly healthier periodontal health status compared to medical students. The mean CPI score for dental students was 1.08 ± 1.1, while for medical students it was 1.22 ± 1.0, both showing statistical significance. These findings align with the higher knowledge scores of dental students and underscore the impact of professional training on oral health outcomes. Literature supports the notion that better education and training in dental students lead to improved periodontal health [20,21].

For dental caries, measured by the DMFT index, the mean score was 1.64 ± 1.66. This relatively low mean score indicates a generally good dental caries status among students. However, significant differences were found when comparing DMFT scores with knowledge and behavior scores. Students with higher knowledge and better oral health behaviors had lower DMFT scores, emphasizing the critical role of education and preventive practices in managing dental caries. Interestingly, attitudes did not show a significant correlation with DMFT scores, suggesting that knowledge and behavior are more direct determinants of dental caries status. These findings are consistent with previous research, which has shown that knowledge and behavior are critical determinants of oral health outcomes. The dominance of the component in the score suggests a high commonness of untreated dental caries among the students, indicating unmet treatment needs [22-24]. The study underscores the importance of positive attitudes and adherence to good oral hygiene behaviors in maintaining oral health.

The findings of this study suggest that enhanced educational programs are needed to improve oral health knowledge and behaviors among medical and dental students. We recommend incorporating more structured oral health education into medical curricula, with an emphasis on preventive care and regular dental visits. Additionally, awareness campaigns should be designed to highlight the importance of proper brushing techniques, toothpaste use, and regular professional dental check-ups. Long-term studies should also be considered to assess the impact of these interventions on oral health outcomes.

Further examination is expected to investigate the intricacies of oral well-being information, perspectives, and ways of behaving in various populaces, considering the limitations of self-reported data and social desirability biases. This knowledge equips them not only to manage their own oral health but also to educate their patients and families effectively. Moreover, targeted interventions to improve professional dental care visits and self-awareness regarding gum health are essential. Educational campaigns focusing on the importance of regular dental check-ups and effective interdental cleaning methods could further enhance oral health outcomes.

The study has some potential limitations, including the use of convenient sampling, which may lead to selection bias and limit the generalizability of the results. The cross-sectional design captures data at a single point in time, restricting insights into changes over time. Reliance on self-reported data for assessing knowledge, attitudes, and behavior can introduce bias, while the limited scope of oral health assessment using the CPI and DMFT indices might overlook other important factors. Additionally, the Cronbach alpha of 0.70 indicates acceptable but not optimal reliability. The geographic focus on students from Taxila may also limit the broader applicability of the findings, and the absence of qualitative data restricts a deeper understanding of underlying factors influencing students' oral health behavior and attitudes.

## Conclusions

The study highlights the crucial relationship of oral health education, attitudes, and behaviors with actual oral health outcomes. The findings suggest that enhancing oral health education, particularly among medical students, could lead to better oral hygiene practices and improved periodontal and dental caries status. Incorporating more comprehensive oral health modules in medical curricula could bridge the knowledge gap and foster positive behavioral changes. By doing so, future healthcare professionals can be better equipped to maintain their oral health and promote similar practices among their patients, ultimately contributing to improved public health outcomes.

As this study focused on medical and dental students, further research is warranted to delve deeper into these issues across various target populations. Additionally, there is a dearth of data on oral healthcare attitudes in Pakistan, underscoring the importance of conducting more detailed studies and establishing a robust database.

To formulate effective strategies for improving dental and oral health in the Pakistani population, it is imperative to gather representative data through additional studies, using reliable and culturally appropriate attitude scales. Such efforts will be instrumental in addressing the oral health needs of diverse communities in Pakistan.

In light of these findings, we recommend the implementation of initiatives to enhance oral/dental health awareness among both medical and non-medical students. Collaborative efforts among oral health professionals are essential to devise a comprehensive strategy for this purpose. Additionally, organizing dental/oral health awareness programs for all students can significantly contribute to improving oral health outcomes. Furthermore, conducting further studies in diverse regions would enable comparative analysis and provide valuable insights for oral health promotion efforts.

## Appendices

                                 Questionnaire

Name (Optional):                                                           Age:    

Gender:                                                                          Student: MBBS/BDS

                                      Section 1

Do you believe that general body wellbeing is related to oral well-being?

·       Yes

·       No

What is the recommended time for brushing your teeth?

·       Less than 1 minute

·       1-2 minutes

·       More than 2 minutes

Can regular brushing protect you from gum bleeding?

·       Yes

·       No

What are some common signs of gum disease?

·       Red or swollen gums

·       Gum bleeding

·       Persistent bad breath

·       Tooth sensitivity

·       Other (please specify)

What is the main purpose of fluoride in oral health?

·       Strengthens tooth enamel

·       Prevents gum disease

·       Reduces sensitivity

·       I don’t know

At what age should a child have their first dental visit?

·       0-1 year

·       1-3 years

·       3-5 years

·       5 years or older

What are the common causes of tooth decay?

·       Poor oral hygiene

·       Frequent consumption of sugary foods

·       Not visiting the dentist regularly

·       Genetic factors

How much toothpaste should be used for brushing?

·       A pea-sized amount

·       Covering the whole toothbrush

·       I don’t know

What are some ways to prevent tooth decay?

·       Brushing twice a day

·       Using fluoride toothpaste

·       Flossing daily

·       Limiting sugary foods and drinks

·       Regular dental check-ups

How do you prevent gum bleeding problems?

·       Regular brushing

·       Flossing

·       Using mouthwash

·       Visiting the dentist regularly

                                              Section 2

To what extent do you agree with the following statements?

(Strongly Disagree, Disagree, Neutral, Agree, Strongly Agree)

a.  I believe oral health is an important part of overall health.

b.  Regular dental checkups are essential for maintaining good oral health.

c.  Oral health problems can significantly impact a person's quality of life.

d.  The general public's knowledge of oral health is very low.

e.  I use floss daily.

f.  Fluoride treatments are important in preventing cavities.

g.  I am comfortable visiting the dentist for a routine checkup.

h.  Cost is the biggest barrier to people seeking dental care.

i.  There is a stigma associated with needing dental treatment.

j.  My education has made me consider oral health to be important.

                                              Section 3

Dental Hygiene Questionnaire for Medical and Dental Students

1.     Toothbrush Replacement

How often do you replace your toothbrush?

o   Every 1-3 months

o   Every 4-6 months

o   Every 7-9 months

o   Less often

2.     Frequency of Tooth Brushing

How many times a day do you brush your teeth?

o   Once

o   Twice

o   More than twice

3.     Duration of Brushing

On average, how much time do you spend brushing your teeth each time?

o   Less than 1 minute

o   1-2 minutes

o   More than 2 minutes

4.     Toothpaste Selection

What is the primary reason for your choice of toothpaste?

o   Price

o   Taste

o   Advertisement

o   Availability at home

o   Other (please specify):                     

5.     Amount of Toothpaste Used

How much toothpaste do you use each time you brush?

o   Pea-sized amount

o   More than pea-sized

6.     Interdental Cleaning

Which interdental cleaning methods do you regularly use?

o   Toothpick

o   Floss

o   Interdental brush

o   None

7.     Professional Dental Care

How often do you visit a dentist for a check-up?

o   Every 6 months

o   Once a year

o   Only when I have a dental problem

o   I don’t visit a dentist regularly

8.     Awareness of Gum Health

Are you aware of any gum-related problems you may have?

o   Yes

o   No

9.     Self-Assessment of Dental Hygiene

How would you rate your overall dental hygiene?

o   Excellent

o   Good

o   Fair

o   Poor

                                            Section 4

Community Periodontal Index

☐  Healthy (no issues)

☐  Bleeding during testing

☐  Calculus detected, but no pocket greater than 3mm

☐  Shallow pockets (4-5mm)

☐  Deep pockets (6mm or more)

Decayed Missing Filled Teeth

Decayed -

Missing -

Filled -

Score (0 to 6) =__________                  
